# OCCUPATIONAL CHARACTERISTICS ASSOCIATED WITH CHRONIC INFLAMMATION: EVIDENCE FROM THE HRS

**DOI:** 10.1093/geroni/igad104.3007

**Published:** 2023-12-21

**Authors:** Theresa Andrasfay, Jennifer Ailshire, Eileen Crimmins

**Affiliations:** California State University San Marcos, San Marcos, California, United States; University of Southern California, Los Angeles, California, United States; University of Southern California, Los Angeles, California, United States

## Abstract

There is substantial evidence from the occupational health, psychology, and environmental health literatures to suggest that inflammation is a biologically plausible mechanism through which physical, psychosocial, and environmental occupational characteristics influence risks of several health conditions at older ages. However, there is limited empirical evidence of the linkages between occupational characteristics and biomarkers of inflammation in representative studies of older adults, precluding a thorough understanding of how working conditions affect the distribution of inflammation among the older population. We use data from 1,997 individuals who were aged 51-60 and working for pay in the 2010 wave of the Health and Retirement Study (HRS) to assess how occupational characteristics are associated with a summary measure of chronic inflammation constructed from seven inflammatory biomarkers included in the 2016 HRS Venous Blood Study. In addition to HRS respondents’ reports of their job characteristics, we use data from the Occupational Information Network (O*NET). We use Poisson models to predict the count of high-risk inflammatory biomarkers from occupational characteristics, controlling for age, sex, race/ethnicity, educational attainment, and use of over-the-counter anti-inflammatory drugs. We find that individuals employed in occupations with higher levels of physical demands, higher levels of harmful workplace exposures, and those working long hours have higher levels of inflammation. Higher levels of autonomy or job control were associated with reduced levels of inflammation. These results suggest that common job conditions may contribute to chronic inflammation at older ages.

